# Supplementary material for: Emojis predict dropouts of remote workers: An empirical study of emoji usage on GitHub
Source: PLoS One. 2022 Jan 26;17(1):e0261262. doi: 10.1371/journal.pone.0261262 (PMC8791473; doi:10.1371/journal.pone.0261262)
Supplement: S1 Appendix — (PDF) [file pone.0261262.s001.pdf]

Appendix

1. Programming language selection We adopt GitHub's definition of "a language." We calculate the popularity of a language based on the number of repositories that identify it as their main languages based on pull request events, pull request, and review comment events in the GHArchive dataset. These languages are (in decreasing popularity): JavaScript, Python, Java, C++, GO, HTML, PHP, Ruby, TypeScript, C#, C, CSS, Shell, Rust, Scala, Swift, Objective-C, PowerShell, Jupyter Notebook, and Kotlin.

2. Regression configurations The independent variables in the first regression (1) are all from category D1 (activity level); the independent variables in the second regression (2) are from D1 and D2, and so on. Finally, in the last model (6), we include all categories of variables (D1-5) as well as the control variables (platform age and programming languages).

3. Regression results We report the full results of the regressions in Table S1, Table S2, and Table S3.

Table S1. OLS Regressions of Emoji Usage (#Emoji Posts) on Working Status.

Dependent Variable: Number of Emoji Posts (Log Scale)								
	(1)	(2)	(3)	(4)	(5)	(6)		(6) (cont.)
const	3.3939***	3.3497***	3.0106***	2.6682***	2.4833***	2.0552***	platform age (log)	0.0373***
	(0.0988)	(0.0989)	(0.0950)	(0.0925)	(0.0905)	(0.0899)		(0.0011)
avg. working hours	0.0322***	0.0335***	0.0355***	0.0468***	0.0240***	0.0334***	C	-0.1719***
	(0.0018)	(0.0018)	(0.0019)	(0.0019)	(0.0018)	(0.0018)		(0.0063)
avg. length of working sessions (in hour)	0.0002	0.0002	0.0006***	0.0004***	0.0003**	0.0002*	CSS	0.0986***
	(0.0002)	(0.0002)	(0.0001)	(0.0001)	(0.0001)	(0.0001)		(0.0069)
length of off segment (threshold = 4)	-0.0366***	-0.0365***	-0.0455***	-0.0393***	-0.0265***	-0.0254***	C#	-0.0230***
	(0.0002)	(0.0002)	(0.0002)	(0.0002)	(0.0002)	(0.0002)		(0.0053)
length of off segment (threshold = 16)	-0.0391***	-0.0390***	-0.0308***	-0.0291***	-0.0324***	-0.0320***	C++	-0.1550***
	(0.0004)	(0.0004)	(0.0004)	(0.0004)	(0.0004)	(0.0004)		(0.0050)
length of off segment (threshold = 32)	-0.0350***	-0.0349***	-0.0269***	-0.0246***	-0.0229***	-0.0231***	GCC	0.5722***
	(0.0007)	(0.0007)	(0.0006)	(0.0006)	(0.0006)	(0.0006)		(-0.0122)
length of off segment (threshold = 64)	-0.0482***	-0.0477***	-0.0351***	-0.0326***	-0.0302***	-0.0293***	Go	-0.0224***
	(0.0011)	(0.0011)	(0.0010)	(0.0010)	(0.0010)	(0.0010)		(-0.0063)
length of off segment (threshold = 128)	-0.0055**	-0.0051**	-0.0105***	-0.0115***	-0.0168***	-0.0153***	HTML	0.0550***
	(0.0024)	(0.0024)	(0.0023)	(0.0023)	(0.0022)	(0.0022)		(-0.0048)
length of off segment (threshold = 256)	0.0322***	0.0329***	0.0233***	0.0255***	0.0104**	0.0145***	Java	-0.1088***
	(0.0046)	(0.0046)	(0.0044)	(0.0042)	(0.0041)	(0.0041)		(-0.0039)
monthly trend of #working days		-0.0206***	-0.0166***	-0.0103***	-0.0101***	-0.0069***	JavaScript	0.1134***
		(0.0017)	(0.0016)	(0.0016)	(0.0015)	(0.0015)		(-0.0029)
monthly trend of *avg.* working hours		0.0130***	0.0132***	0.0129***	0.0098***	0.0117***	Jupyter Notebook	-0.0953***
		(0.0016)	(0.0015)	(0.0015)	(0.0014)	(0.0014)		(-0.0132)
monthly trend of *avg.* length of working sessions		0.0002	-0.0019	-0.0012	-0.0001	0.0019	Objective C	0.0169
		(0.0015)	(0.0014)	(0.0014)	(0.0013)	(0.0013)		(-0.0111)
monthly trend of #working sessions with >1 event		-0.0068***	-0.0101***	-0.0099***	-0.0077***	-0.0068***	PHP	0.0170***
		(0.0017)	(0.0016)	(0.0016)	(0.0015)	(0.0015)		(-0.0050)
monthly trend of #posts		0.0059***	0.0128***	0.0085***	0.0105***	0.0113***	PowerShell	0.4798***
		(0.0013)	(0.0013)	(0.0012)	(0.0012)	(0.0012)		(-0.0078)
prop. pull request events			0.7475***	0.8098***	0.2979***	0.2079***	Python	-0.0658***
			(0.0164)	(0.0167)	(0.0166)	(0.0166)		(-0.0037)
prop. push events			-0.2224***	-0.2253***	-0.3261***	-0.3165***	Ruby	0.1613***
			(0.0048)	(0.0047)	(0.0047)	(0.0047)		(-0.0060)
prop. working sessions with pull request events			0.0288***	0.0718***	0.4902***	0.5022***	Rust	0.1202***
			(0.0074)	(0.0074)	(0.0077)	(0.0077)		(-0.0136)
prop. working days with comment events			0.5437***	0.2777***	0.3839***	0.4069***	Scala	-0.0681***
			(0.0034)	(0.0045)	(0.0045)	(0.0045)		(-0.0139)
prop. working days with issue events			0.1998***	0.2637***	0.4348***	0.3983***	Shell	-0.0205***
			(0.0038)	(0.0049)	(0.0049)	(0.0049)		(-0.0079)
prop. pull requests				-0.1556***	-0.1428***	-0.1269***	Swift	0.2226***
				(0.0051)	(0.0050)	(0.0049)		(-0.0098)
prop. issue comments				0.0902***	0.0223***	0.0176***	TypeScript	0.1006***
				(0.0053)	(0.0052)	(0.0051)		(-0.0059)
prop. pull request comments				0.6779***	0.5859***	0.5622***		
				(0.0071)	(0.0070)	(0.0069)		
prop. pull request review comments				0.7706***	0.6380***	0.6093***		
				(0.0079)	(0.0078)	(0.0078)		
prop. commit comments				-0.0087	0.0355***	0.0559***		
				(0.0083)	(0.0081)	(0.0080)		
entropy of weekdays					0.3188***	0.3030***		
					(0.0021)	(0.0021)		
variables about backgrounds						see right		
# Obs.	529616	529616	529616	529616	529616	529616		
R-squared	0.3548	0.3552	0.4140	0.4466	0.4704	0.4845		
Adj. R-squared	0.3549	0.3552	0.4141	0.4466	0.4704	0.4846		

Notes: Standard errors in parentheses.
Significant at the: *** 1%, ** 5%, or * 10% level.

Table S2. OLS Regressions of Emoji Usage (*prop.* Emoji Posts) on Working Status.

Dependent Variable: Proportion of Emoji Posts								
	(1)	(2)	(3)	(4)	(5)	(6)		(6) (cont.)
const	0.5533***	0.5617***	0.3244***	0.2649***	0.2867***	0.1903***	platform age (log)	0.0032***
	(0.0313)	(0.0313)	(0.0313)	(0.0311)	(0.0310)	(0.0301)		(0.0004)
avg. working hours	-0.0223***	-0.0227***	-0.0068***	-0.0037***	-0.0010	0.0024***	C	-0.0348***
	(0.0006)	(0.0006)	(0.0006)	(0.0006)	(0.0006)	(0.0006)		(0.0021)
avg. length of working sessions (in hour)	0.0003***	0.0003***	0.0003***	0.0002***	0.0002***	0.0002***	CSS	0.0227***
	(0.0000)	(0.0000)	(0.0000)	(0.0000)	(0.0000)	(0.0000)		(0.0023)
length of off segment (threshold = 4)	0.0026***	0.0026***	0.0023***	0.0030***	0.0015***	0.0016***	C#	-0.0032*
	(0.0001)	(0.0001)	(0.0001)	(0.0001)	(0.0001)	(0.0001)		(0.0018)
length of off segment (threshold = 16)	0.0001	0.0001	0.0001	0.0003**	0.0007***	0.0008***	C++	-0.0324***
	(0.0001)	(0.0001)	(0.0001)	(0.0001)	(0.0001)	(0.0001)		(0.0017)
length of off segment (threshold = 32)	-0.0005**	-0.0006***	-0.0006***	-0.0003*	-0.0005***	-0.0006***	GCC	0.4173***
	(0.0002)	(0.0002)	(0.0002)	(0.0002)	(0.0002)	(0.0002)		(0.0041)
length of off segment (threshold = 64)	-0.0026***	-0.0027***	-0.0020***	-0.0014***	-0.0017***	-0.0014***	Go	-0.0087***
	(0.0003)	(0.0003)	(0.0003)	(0.0003)	(0.0003)	(0.0003)		(0.0021)
length of off segment (threshold = 128)	-0.0087***	-0.0088***	-0.0047***	-0.0037***	-0.0031***	-0.0022***	HTML	0.0119***
	(0.0008)	(0.0008)	(0.0008)	(0.0008)	(0.0008)	(0.0007)		(0.0016)
length of off segment (threshold = 256)	-0.0074***	-0.0074***	-0.0014	-0.0003	0.0015	0.0029**	Java	-0.0328***
	(0.0014)	(0.0014)	(0.0014)	(0.0014)	(0.0014)	(0.0014)		(0.0013)
monthly trend of #working days		0.0027***	0.0022***	0.0028***	0.0028***	0.0014***	JavaScript	0.0140***
		(0.0005)	(0.0005)	(0.0005)	(0.0005)	(0.0005)		(0.0010)
monthly trend of *avg.* working hours		0.0022***	0.0022***	0.0022***	0.0025***	0.0014***	Jupyter Notebook	-0.0308***
		(0.0005)	(0.0005)	(0.0005)	(0.0005)	(0.0005)		(0.0044)
monthly trend of *avg.* length of working sessions		-0.0023***	-0.0022***	-0.0023***	-0.0024***	-0.0013***	Objective C	-0.0043
		(0.0005)	(0.0005)	(0.0005)	(0.0005)	(0.0004)		(0.0037)
monthly trend of #working sessions with >1 event		-0.0061***	-0.0055***	-0.0053***	-0.0056***	-0.0037***	PHP	-0.0151***
		(0.0005)	(0.0005)	(0.0005)	(0.0005)	(0.0005)		(0.0017)
monthly trend of #posts		0.0087***	0.0081***	0.0078***	0.0075***	0.0064***	PowerShell	0.4256***
		(0.0004)	(0.0004)	(0.0004)	(0.0004)	(0.0004)		(0.0026)
prop. pull request events			-0.1669***	-0.1606***	-0.1003***	-0.1090***	Python	-0.0231***
			(0.0054)	(0.0056)	(0.0057)	(0.0056)		(0.0012)
prop. push events			-0.0569***	-0.0617***	-0.0498***	-0.0496***	Ruby	0.0268***
			(0.0016)	(0.0016)	(0.0016)	(0.0016)		(0.0020)
prop. working sessions with pull request events			-0.0072***	0.0055**	-0.0438***	-0.0476***	Rust	-0.0003
			(0.0025)	(0.0025)	(0.0026)	(0.0026)		(0.0046)
prop. working days with comment events			-0.0679***	-0.0843***	-0.0968***	-0.0871***	Scala	-0.0283***
			(0.0011)	(0.0015)	(0.0015)	(0.0015)		(0.0047)
prop. working days with issue events			0.0267***	0.0052***	-0.0150***	-0.0487***	Shell	-0.0082***
			(0.0013)	(0.0016)	(0.0017)	(0.0016)		(0.0026)
prop. pull requests				-0.0637***	-0.0652***	-0.0606***	Swift	0.0466***
				(0.0017)	(0.0017)	(0.0017)		(0.0033)
prop. issue comments				-0.0479***	-0.0399***	-0.0369***	TypeScript	0.0083***
				(0.0018)	(0.0018)	(0.0017)		(0.0020)
prop. pull request comments				0.1339***	0.1447***	0.1384***		
				(0.0024)	(0.0024)	(0.0023)		
prop. pull request review comments				-0.0363***	-0.0206***	-0.0196***		
				(0.0027)	(0.0027)	(0.0026)		
prop. commit comments				-0.0258***	-0.0310***	-0.0284***		
				(0.0028)	(0.0028)	(0.0027)		
entropy of weekdays					-0.0376***	-0.0337***		
					(0.0007)	(0.0007)		
variables about backgrounds						see right		
# Obs.	529616	529616	529616	529616	529616	529616		
R-squared	0.0161	0.0176	0.0340	0.0538	0.0588	0.1255		
Adj. R-squared	0.0161	0.0176	0.0340	0.0538	0.0588	0.1255		

Notes: Standard errors in parentheses.
Significant at the: *** 1%, ** 5%, or * 10% level.

4. Precision-recall curve Fig S1 presents the precision-recall curve of GBDT for the 30 datasets with the $k = 5$ activity level (by number of working days). This curve demonstrates the precision values for units ranked by their predicted probability of dropping out.

5. Dropout ratio vs. programming languages To demonstrate the relation between the primary programming language and the working status outcome, we plot the distribution of dropout rates in the 20 most popular programming languages in Fig S2. Variance could be observed among programming languages from both the developers who used emojis and the others in 2018.

Table S3. OLS Regressions of Emoji Usage (*prop.* Emotional Emojis) on Working Status.

	Dependent Variable: Prop. emotional emojis							
	(1)	(2)	(3)	(4)	(5)	(6)		(6) (cont.)
const	1.4479***	1.4788***	1.2302***	1.1711***	1.2646***	0.9679***	platform age (log)	0.0030**
	(0.0693)	(0.0693)	(0.0697)	(0.0699)	(0.0706)	(0.0710)		(0.0012)
avg. working hours	-0.0586***	-0.0599***	-0.0444***	-0.0384***	-0.0403***	-0.0294***	C	-0.0103*
	(0.0017)	(0.0017)	(0.0018)	(0.0018)	(0.0018)	(0.0018)		(0.0062)
avg. length of working sessions	0.0002**	0.0002**	0.0002	0.0001	0.0001	0.0001	CSS	-0.0014
	(0.0001)	(0.0001)	(0.0001)	(0.0001)	(0.0001)	(0.0001)		(0.0061)
length of off segment (threshold = 1)	0.0029***	0.0028***	0.0017***	0.0019***	0.0002	-0.0006*	C#	0.1203***
	(0.0002)	(0.0002)	(0.0002)	(0.0002)	(0.0003)	(0.0003)		(0.0051)
length of off segment (threshold = 4)	-0.0019***	-0.0019***	-0.0019***	-0.0021***	-0.0023***	-0.0016***	C++	-0.0209***
	(0.0002)	(0.0002)	(0.0002)	(0.0002)	(0.0002)	(0.0002)		(0.0048)
length of off segment (threshold = 16)	-0.0021***	-0.0021***	-0.0018***	-0.0018***	-0.0017***	-0.0013***	GCC	0.6059***
	(0.0003)	(0.0003)	(0.0003)	(0.0003)	(0.0003)	(0.0003)		(0.0088)
length of off segment (threshold = 32)	-0.0002	-0.0004	-0.0001	-0.0002	-0.0002	-0.0000	Go	-0.0027
	(0.0004)	(0.0004)	(0.0004)	(0.0004)	(0.0004)	(0.0004)		(0.0052)
length of off segment (threshold = 64)	-0.0059***	-0.0062***	-0.0049***	-0.0048***	-0.0050***	-0.0041***	HTML	0.0196***
	(0.0007)	(0.0007)	(0.0007)	(0.0007)	(0.0007)	(0.0007)		(0.0046)
length of off segment (threshold = 128)	-0.0113***	-0.0115***	-0.0075***	-0.0062***	-0.0066***	-0.0043***	Java	-0.0090**
	(0.0016)	(0.0016)	(0.0016)	(0.0016)	(0.0016)	(0.0016)		(0.0040)
length of off segment (threshold = 256)	-0.0210***	-0.0214***	-0.0155***	-0.0128***	-0.0133***	-0.0091***	JavaScript	0.0027
	(0.0030)	(0.0030)	(0.0030)	(0.0030)	(0.0030)	(0.0030)		(0.0026)
monthly trend of #events		0.0058***	0.0062***	0.0066***	0.0065***	0.0042**	Jupyter Notebook	0.0076
		(0.0018)	(0.0018)	(0.0018)	(0.0018)	(0.0018)		(0.0136)
monthly trend of #working days		0.0098***	0.0081***	0.0076***	0.0076***	0.0059***	Objective C	-0.0031
		(0.0015)	(0.0015)	(0.0015)	(0.0015)	(0.0015)		(0.0103)
monthly trend of *avg.* working hours		0.0061***	0.0054***	0.0055***	0.0055***	0.0042***	PHP	-0.0205***
		(0.0013)	(0.0013)	(0.0013)	(0.0013)	(0.0013)		(0.0046)
monthly trend of *avg.* length of working sessions		-0.0045***	-0.0039***	-0.0039***	-0.0039***	-0.0025**	PowerShell	0.3107***
		(0.0012)	(0.0012)	(0.0012)	(0.0012)	(0.0011)		(0.0060)
monthly trend of #working sessions with >1 event		-0.0170***	-0.0151***	-0.0150***	-0.0149***	-0.0117***	Python	-0.0020
		(0.0016)	(0.0016)	(0.0016)	(0.0016)	(0.0015)		(0.0035)
monthly trend of #posts		0.0139***	0.0125***	0.0126***	0.0126***	0.0113***	Ruby	-0.0063
		(0.0012)	(0.0012)	(0.0012)	(0.0012)	(0.0012)		(0.0049)
prop. comment events			-0.0968***	-0.0680***	-0.0696***	-0.0491***	Rust	0.0432***
			(0.0044)	(0.0058)	(0.0058)	(0.0059)		(0.0102)
prop. working days with issue events			0.0595***	0.0038	-0.0061	-0.0781***	Scala	-0.0157
			(0.0042)	(0.0054)	(0.0055)	(0.0056)		(0.0116)
prop. pull request events			-0.3498***	-0.2256***	-0.2212***	-0.2143***	Shell	-0.0024
			(0.0103)	(0.0137)	(0.0137)	(0.0137)		(0.0070)
prop. push events			-0.0532***	-0.0443***	-0.0402***	-0.0307***	Swift	0.0463***
			(0.0050)	(0.0051)	(0.0051)	(0.0050)		(0.0082)
prop. commit comments				-0.1130***	-0.1160***	-0.0979***	TypeScript	0.0142***
				(0.0110)	(0.0110)	(0.0109)		(0.0051)
prop. issue comments				-0.0459***	-0.0430***	-0.0228***		
				(0.0060)	(0.0060)	(0.0059)		
prop. pull request comments				-0.0890***	-0.0900***	-0.0857***		
				(0.0070)	(0.0070)	(0.0069)		
prop. pull requests				-0.0811***	-0.0840***	-0.0739***		
				(0.0065)	(0.0065)	(0.0064)		
prop. pull request review comments				-0.1117***	-0.1091***	-0.1056***		
				(0.0076)	(0.0076)	(0.0076)		
entropy of weekdays					-0.0298***	-0.0034		
					(0.0033)	(0.0033)		
variables about backgrounds						see right		
# Obs.	264808	264808	264808	264808	264808	264808		
R-squared	0.0064	0.0084	0.0159	0.0173	0.0176	0.0443		
	0.0064	0.0084	0.0160	0.0174	0.0177	0.0444		

Notes: Standard errors in parentheses.
Significant at the: *** 1%, ** 5%, or * 10% level.

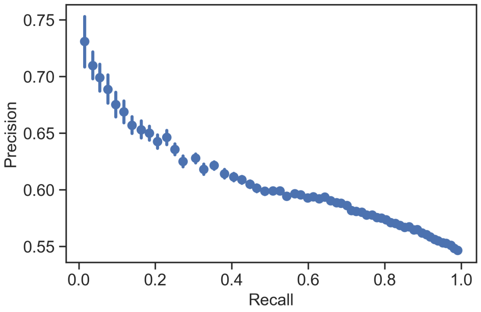

Fig S1. Precision-recall curve for top 5% active users (by #working days). The values of recall and precision at different threshold levels are binned for the 30 sampled datasets.

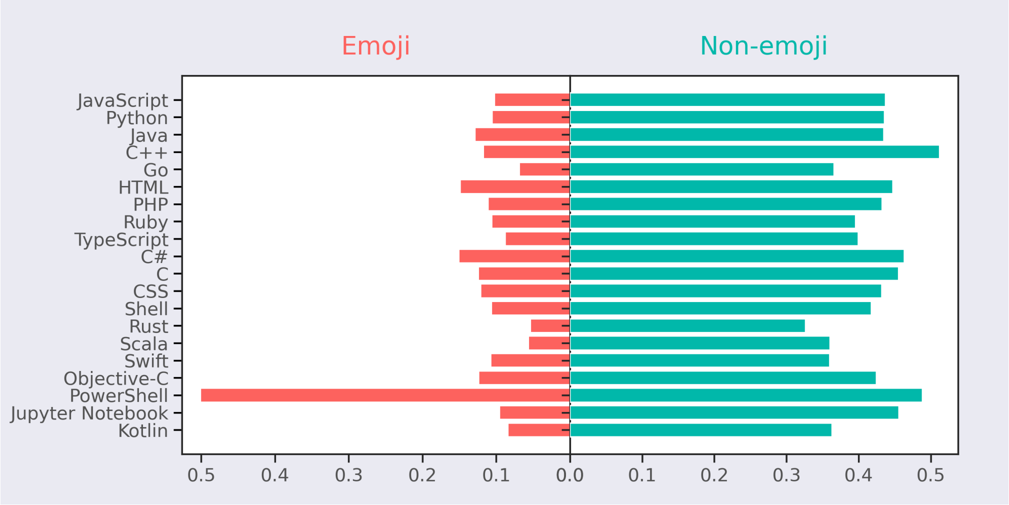

Fig S2. Dropout ratio for developers with different primary programming languages. Emoji: developers who used at least one emoji in 2018. Non-emoji: developers who did not use any emoji in 2018.
