# Supplementary figures and images for: Emojis predict dropouts of remote workers: An empirical study of emoji usage on GitHub
Source: PLoS One. 2022 Jan 26;17(1):e0261262. doi: 10.1371/journal.pone.0261262 (PMC8791473; doi:10.1371/journal.pone.0261262)

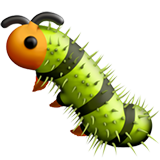

Supplement: S1 File — (ZIP) [file pone.0261262.s002.zip › emoji_images/bug_1f41b.png]

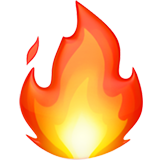

Supplement: S1 File — (ZIP) [file pone.0261262.s002.zip › emoji_images/fire_1f525.png]

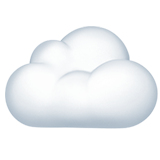

Supplement: S1 File — (ZIP) [file pone.0261262.s002.zip › emoji_images/cloud_2601.png]

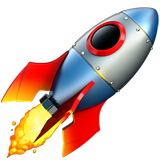

Supplement: S1 File — (ZIP) [file pone.0261262.s002.zip › emoji_images/rocket_1f680.png]

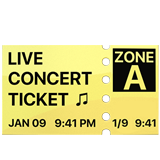

Supplement: S1 File — (ZIP) [file pone.0261262.s002.zip › emoji_images/ticket_1f3ab.png]

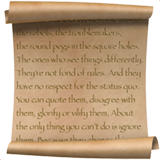

Supplement: S1 File — (ZIP) [file pone.0261262.s002.zip › emoji_images/scroll_1f4dc.png]

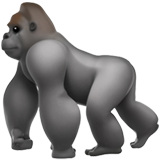

Supplement: S1 File — (ZIP) [file pone.0261262.s002.zip › emoji_images/gorilla_1f98d.png]

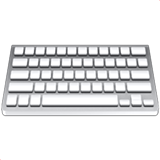

Supplement: S1 File — (ZIP) [file pone.0261262.s002.zip › emoji_images/keyboard_2328.png]

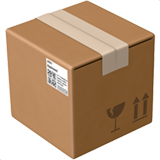

Supplement: S1 File — (ZIP) [file pone.0261262.s002.zip › emoji_images/package_1f4e6.png]

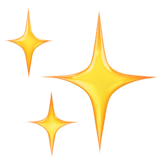

Supplement: S1 File — (ZIP) [file pone.0261262.s002.zip › emoji_images/sparkles_2728.png]

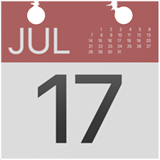

Supplement: S1 File — (ZIP) [file pone.0261262.s002.zip › emoji_images/calendar_1f4c5.png]

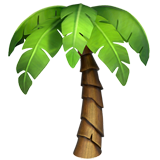

Supplement: S1 File — (ZIP) [file pone.0261262.s002.zip › emoji_images/palm-tree_1f334.png]

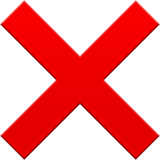

Supplement: S1 File — (ZIP) [file pone.0261262.s002.zip › emoji_images/cross-mark_274c.png]

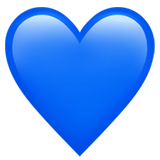

Supplement: S1 File — (ZIP) [file pone.0261262.s002.zip › emoji_images/blue-heart_1f499.png]

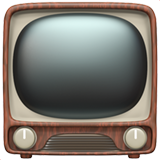

Supplement: S1 File — (ZIP) [file pone.0261262.s002.zip › emoji_images/television_1f4fa.png]

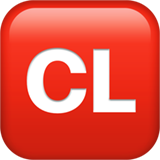

Supplement: S1 File — (ZIP) [file pone.0261262.s002.zip › emoji_images/squared-cl_1f191.png]

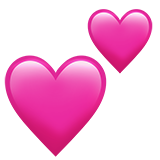

Supplement: S1 File — (ZIP) [file pone.0261262.s002.zip › emoji_images/two-hearts_1f495.png]

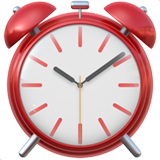

Supplement: S1 File — (ZIP) [file pone.0261262.s002.zip › emoji_images/alarm-clock_23f0.png]

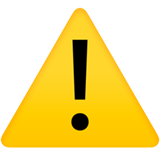

Supplement: S1 File — (ZIP) [file pone.0261262.s002.zip › emoji_images/warning-sign_26a0.png]

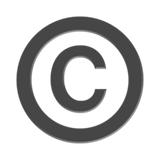

Supplement: S1 File — (ZIP) [file pone.0261262.s002.zip › emoji_images/copyright-sign_a9.png]

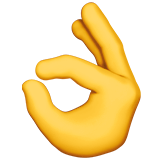

Supplement: S1 File — (ZIP) [file pone.0261262.s002.zip › emoji_images/ok-hand-sign_1f44c.png]

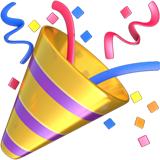

Supplement: S1 File — (ZIP) [file pone.0261262.s002.zip › emoji_images/party-popper_1f389.png]

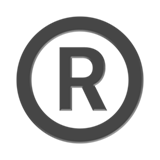

Supplement: S1 File — (ZIP) [file pone.0261262.s002.zip › emoji_images/registered-sign_ae.png]

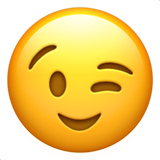

Supplement: S1 File — (ZIP) [file pone.0261262.s002.zip › emoji_images/winking-face_1f609.png]

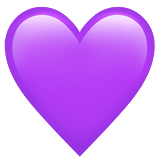

Supplement: S1 File — (ZIP) [file pone.0261262.s002.zip › emoji_images/purple-heart_1f49c.png]

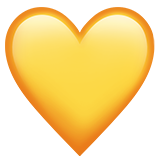

Supplement: S1 File — (ZIP) [file pone.0261262.s002.zip › emoji_images/yellow-heart_1f49b.png]

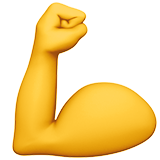

Supplement: S1 File — (ZIP) [file pone.0261262.s002.zip › emoji_images/flexed-biceps_1f4aa.png]

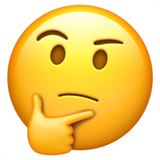

Supplement: S1 File — (ZIP) [file pone.0261262.s002.zip › emoji_images/thinking-face_1f914.png]

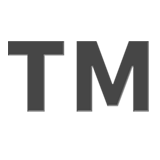

Supplement: S1 File — (ZIP) [file pone.0261262.s002.zip › emoji_images/trade-mark-sign_2122.png]

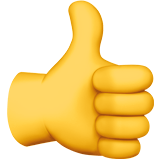

Supplement: S1 File — (ZIP) [file pone.0261262.s002.zip › emoji_images/thumbs-up-sign_1f44d.png]

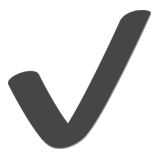

Supplement: S1 File — (ZIP) [file pone.0261262.s002.zip › emoji_images/heavy-check-mark_2714.png]

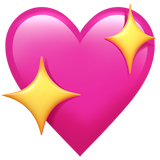

Supplement: S1 File — (ZIP) [file pone.0261262.s002.zip › emoji_images/sparkling-heart_1f496.png]

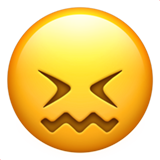

Supplement: S1 File — (ZIP) [file pone.0261262.s002.zip › emoji_images/confounded-face_1f616.png]

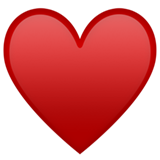

Supplement: S1 File — (ZIP) [file pone.0261262.s002.zip › emoji_images/black-heart-suit_2665.png]

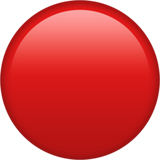

Supplement: S1 File — (ZIP) [file pone.0261262.s002.zip › emoji_images/large-red-circle_1f534.png]

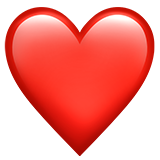

Supplement: S1 File — (ZIP) [file pone.0261262.s002.zip › emoji_images/heavy-black-heart_2764.png]

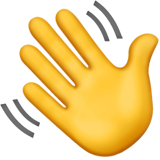

Supplement: S1 File — (ZIP) [file pone.0261262.s002.zip › emoji_images/waving-hand-sign_1f44b.png]

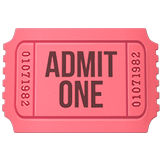

Supplement: S1 File — (ZIP) [file pone.0261262.s002.zip › emoji_images/admission-tickets_1f39f.png]

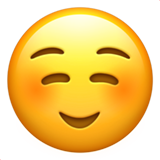

Supplement: S1 File — (ZIP) [file pone.0261262.s002.zip › emoji_images/white-smiling-face_263a.png]

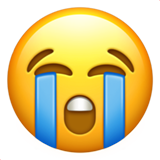

Supplement: S1 File — (ZIP) [file pone.0261262.s002.zip › emoji_images/loudly-crying-face_1f62d.png]

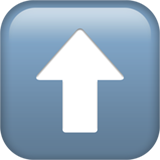

Supplement: S1 File — (ZIP) [file pone.0261262.s002.zip › emoji_images/upwards-black-arrow_2b06.png]

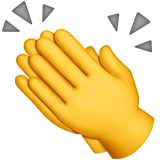

Supplement: S1 File — (ZIP) [file pone.0261262.s002.zip › emoji_images/clapping-hands-sign_1f44f.png]

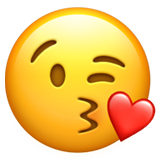

Supplement: S1 File — (ZIP) [file pone.0261262.s002.zip › emoji_images/face-throwing-a-kiss_1f618.png]

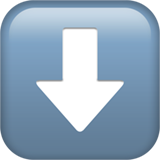

Supplement: S1 File — (ZIP) [file pone.0261262.s002.zip › emoji_images/downwards-black-arrow_2b07.png]

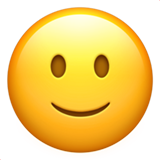

Supplement: S1 File — (ZIP) [file pone.0261262.s002.zip › emoji_images/slightly-smiling-face_1f642.png]

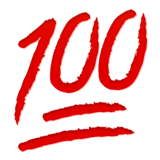

Supplement: S1 File — (ZIP) [file pone.0261262.s002.zip › emoji_images/hundred-points-symbol_1f4af.png]

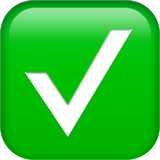

Supplement: S1 File — (ZIP) [file pone.0261262.s002.zip › emoji_images/white-heavy-check-mark_2705.png]

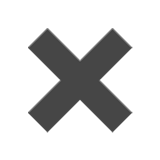

Supplement: S1 File — (ZIP) [file pone.0261262.s002.zip › emoji_images/heavy-multiplication-x_2716.png]

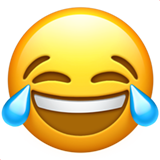

Supplement: S1 File — (ZIP) [file pone.0261262.s002.zip › emoji_images/face-with-tears-of-joy_1f602.png]

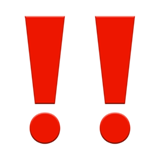

Supplement: S1 File — (ZIP) [file pone.0261262.s002.zip › emoji_images/double-exclamation-mark_203c.png]

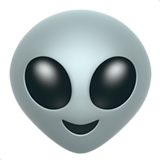

Supplement: S1 File — (ZIP) [file pone.0261262.s002.zip › emoji_images/extraterrestrial-alien_1f47d.png]

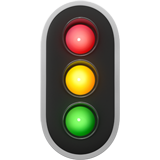

Supplement: S1 File — (ZIP) [file pone.0261262.s002.zip › emoji_images/vertical-traffic-light_1f6a6.png]

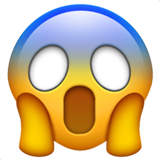

Supplement: S1 File — (ZIP) [file pone.0261262.s002.zip › emoji_images/face-screaming-in-fear_1f631.png]

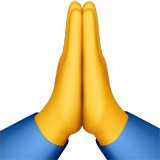

Supplement: S1 File — (ZIP) [file pone.0261262.s002.zip › emoji_images/person-with-folded-hands_1f64f.png]

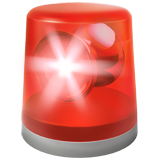

Supplement: S1 File — (ZIP) [file pone.0261262.s002.zip › emoji_images/police-cars-revolving-light_1f6a8.png]

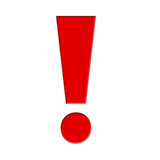

Supplement: S1 File — (ZIP) [file pone.0261262.s002.zip › emoji_images/heavy-exclamation-mark-symbol_2757.png]

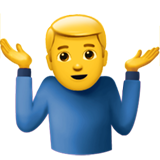

Supplement: S1 File — (ZIP) [file pone.0261262.s002.zip › emoji_images/man-shrugging_1f937-200d-2642-fe0f.png]

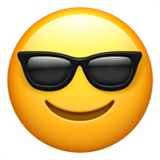

Supplement: S1 File — (ZIP) [file pone.0261262.s002.zip › emoji_images/smiling-face-with-sunglasses_1f60e.png]

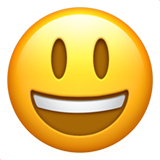

Supplement: S1 File — (ZIP) [file pone.0261262.s002.zip › emoji_images/smiling-face-with-open-mouth_1f603.png]

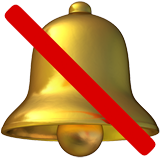

Supplement: S1 File — (ZIP) [file pone.0261262.s002.zip › emoji_images/bell-with-cancellation-stroke_1f515.png]

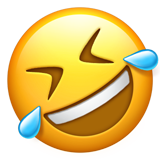

Supplement: S1 File — (ZIP) [file pone.0261262.s002.zip › emoji_images/rolling-on-the-floor-laughing_1f923.png]

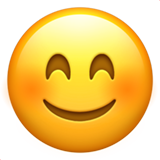

Supplement: S1 File — (ZIP) [file pone.0261262.s002.zip › emoji_images/smiling-face-with-smiling-eyes_1f60a.png]

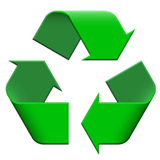

Supplement: S1 File — (ZIP) [file pone.0261262.s002.zip › emoji_images/black-universal-recycling-symbol_267b.png]

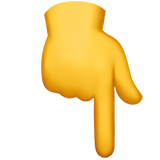

Supplement: S1 File — (ZIP) [file pone.0261262.s002.zip › emoji_images/white-down-pointing-backhand-index_1f447.png]

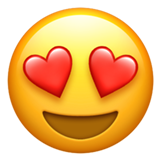

Supplement: S1 File — (ZIP) [file pone.0261262.s002.zip › emoji_images/smiling-face-with-heart-shaped-eyes_1f60d.png]

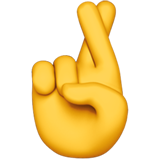

Supplement: S1 File — (ZIP) [file pone.0261262.s002.zip › emoji_images/hand-with-index-and-middle-fingers-crossed_1f91e.png]

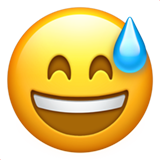

Supplement: S1 File — (ZIP) [file pone.0261262.s002.zip › emoji_images/smiling-face-with-open-mouth-and-cold-sweat_1f605.png]

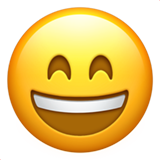

Supplement: S1 File — (ZIP) [file pone.0261262.s002.zip › emoji_images/smiling-face-with-open-mouth-and-smiling-eyes_1f604.png]

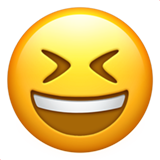

Supplement: S1 File — (ZIP) [file pone.0261262.s002.zip › emoji_images/smiling-face-with-open-mouth-and-tightly-closed-eyes_1f606.png]
